# The Effect of Neoadjuvant Therapies for Patients with Locally Advanced Gastric Cancer: A Propensity Score Matching Study

**DOI:** 10.7150/jca.46847

**Published:** 2021-01-01

**Authors:** Tongbo Wang, Yingtai Chen, Lulu Zhao, Hong Zhou, Chaorui Wu, Xiaojie Zhang, Aiping Zhou, Jing Jin, Dongbing Zhao

**Affiliations:** 1 Department of Pancreatic and Gastric Surgical Oncology, National Cancer Center/ National Clinical Research for Cancer/Cancer Hospital, Chinese Academy of Medical Sciences and Peking Union Medical College, Beijing 100021, China.; 2 Department of Medical Oncology, National Cancer Center/National Clinical Research for Cancer/Cancer Hospital, Chinese Academy of Medical Sciences and Peking Union Medical College, Beijing 100021, China.; 3 Department of Radiation Oncology, National Cancer Center/ National Clinical Research for Cancer/Cancer Hospital, Chinese Academy of Medical Sciences and Peking Union Medical College, Beijing 100021, China.

**Keywords:** locally advanced gastric cancer, neoadjuvant therapies, adjuvant chemotherapy, neoadjuvant chemotherapy, neoadjuvant chemoradiation

## Abstract

**Background:** The aim of this study was to evaluate the effect of neoadjuvant therapies (NAT) on patients with locally advanced gastric cancer (LAGC).

**Methods:** This study retrospectively analyzed LAGC patients treated at the China National Cancer Center between October 2006 and December 2018. All patients included were divided into two groups, NAT followed by surgery (NAT-Surgery) and adjuvant chemotherapy following surgery (Surgery-ACT). Subgroup analysis compared between patients underwent either neoadjuvant chemotherapy (nCT) or neoadjuvant chemoradiation (nCRT) was conducted. Propensity score matching (PSM) was implemented to reduce selection bias.

**Results:** In total, 2779 patients were included in this study (494 of NAT-Surgery group and 2285 of Surgery-ACT group). After PSM, the patients in NAT-Surgery group had a significantly longer overall survival (OS) than patients in Surgery-ACT group (*P*<0.001). Subgroup analysis revealed that grade 3 or 4 adverse events were more frequently observed in nCRT group during neoadjuvant treatment (52.0% in nCRT group vs. 34.0% in nCT group, *P*=0.010). Pathological complete response (pCR) being achieved in 17.0% after nCRT versus 4.0% after nCT (*P*<0.001). Patients of the nCRT group obtained better disease-free survival (DFS, *P*=0.024) and local-recurrence-free survival (LRFS, *P*=0.014) than patients in nCT group, while there was no significant difference in OS between the two groups.

**Conclusions:** In conclusion, NAT improved survival outcomes among LAGC patients over surgery followed by adjuvant chemotherapy. In comparison with nCT, nCRT resulted in higher pCR rate, better DFS and LRFS, without significantly affecting OS.

## Introduction

Gastric cancer is the third leading cause of cancer-related mortality worldwide 1. At the time of diagnosis, more than 50% of patients with gastric cancer have locally advanced disease 2. Despite radical resection, most patients with locally advanced gastric cancer (LAGC) develop recurrence and the 5-year survival rates remain below 50% 3, 4. Thus, the need to find a way to improve survival in locally advanced gastric cancer (LAGC) patients has led to the creation of the so-called “multimodality” strategy that included pre- and post-operative approaches.

Neoadjuvant and adjuvant therapies are generally accepted to improve disease-free survival (DFS) and overall survival (OS) for LAGC patients who have undergone curative resection, however, little consensus exists on the optimum strategy. Perioperative chemotherapy additional to R0 resection is the most popular strategy in Europe, whereas in the USA it is postoperative chemoradiotherapy, and in Asia it is postoperative chemotherapy 5. These recommendations are based on US 0116 trial 6, MAGIC trail 7 and FNCLCC/FFCD ACCORD trial 3, which showed survival benefits with postoperative chemoradiotherapy and perioperative chemotherapy, respectively, compared with surgery alone. In Asia, the recommendation for postoperative chemotherapy is based on ACTS-GC trial 8 and CLASSIC trial 9, which showed a survival benefit with adjuvant chemotherapy after D2 gastrectomy compared with surgery alone. However, there are limited studies that compare the outcomes between these treatment options despite there are some ongoing trials such as NCT01515748 and phase III TOPGEAR trail. In addition, adding radiation therapy to neoadjuvant therapy for LAGC is still considered investigational.

As such, we conducted this retrospective study with the primary aim of comparing the long-term survival between patients of LAGC treated with and without neoadjuvant therapies (NAT). The secondary aim of this study was to assess the short-term and long-term outcomes regarding the two NAT concepts, neoadjuvant chemotherapy (nCT) and neoadjuvant chemoradiation (nCRT). The evaluation was performed by propensity score matching (PSM).

## Methods

### Patients

From October 2006 to December 2018, patients with clinical stage II-III gastric cancer treated with curative gastrectomy following neoadjuvant therapy (NAT-Surgery) or curative gastrectomy followed by adjuvant chemotherapy (Surgery-ACT) at China National Cancer Center were retrospectively reviewed. Inclusion criteria were as follows: presence of locally advanced (cT2-4a and/or N+) gastric adenocarcinoma according to the TNM stage system (American Joint Committee on Cancer, 8th edition); absence of distant metastases confirmed by clinical examination and imaging techniques; no other primary malignancy in the previous 5 years; an Eastern Cooperative Oncology Group performance status (PS) of 0-1. All study procedures were approved by the Institutional Review Board at the China National Cancer Center.

Pretreatment patient evaluation included clinical examination, blood tests, upper gastrointestinal endoscopy ± endoscopic ultrasound (EUS), enhanced chest-abdominal-pelvic enhanced computed tomography-scan (CT-scan) to determine the extent of the disease.

### Neoadjuvant therapies

Neoadjuvant therapies included of neoadjuvant chemotherapy (nCT group) and neoadjuvant chemoradiation (nCRT group). All the patients were evaluated by a multidisciplinary team including gastrointestinal surgeons, medical oncologists, radiation oncologists and radiologists. Based on the patient's age, comorbidities, clinical TNM stage, and a pretreatment evaluation consisted of physical examination, complete blood count, hepatic function, serum tumor marker assessment and electrocardiogram, the most suitable therapeutic strategy was recommended.

#### Neoadjuvant chemotherapy

All patients from nCT group received 4-6 cycles of chemotherapy at the Department of Medical Oncology. Chemotherapy regimens consisted of SOX (S-1/oxaliplatin), FOLFOX (oxaliplatin/leucovorin/fluorouracil) and taxane-based therapy of FOLT (fluorouracil/leucovorin/oxaliplatin/docetaxel) and DOS (S-1/oxaliplatin/docetaxel).

#### Neoadjuvant chemoradiation

For patients from nCRT group, neoadjuvant therapeutic settings included induction chemotherapy followed by concurrent chemoradiation (induction CT+CCRT) and CCRT alone. Induction CT was based on the regimen of SOX as described above for 2-4 cycles. For CCRT planning, patients received two irradiation dose levels of planning target volume (PTV) and PTV boost of 40.04 and 45.1 Gy 22 fractions plus S-1 (80 mg/m^2^/d) administrated on radiotherapy days. Radiotherapy was delivered to the entire stomach and draining regional lymph nodes using three-dimensional (3D) conformal techniques, intensity-modulated radiotherapy (IMRT).

CT-scan and /or positron emission tomography (PET) scan was performed during the therapy to monitor the clinical response. The tumor response was classified according to the *Response Evaluation Criteria in Solid Tumors* (RECIST) 10 and the adverse events of chemotherapy were assessed according to the *National Cancer Institute-Common Terminology Criteria for Adverse Events* (NCI-CTCAE version 4.0).

### Surgery

For all patients included in the present study, the type of gastrectomy, totally or sub-totally, was determined by the size and location of the primary tumor. For patients underwent NAT, Surgery was scheduled 4-6 weeks after the completion of neoadjuvant treatment. D2 lymphadenectomy with spleen preservation was recommended for all patients. Postoperative complications were defined according to the Clavien-Dindo classification 11.

### Pathological assessment

All resected specimens were reviewed by two pathologists to evaluate the pathological response to neoadjuvant treatment and the extent of the residual disease. The pathological tumor staging was determined according to the American Joint Committee on Cancer (AJCC) TNM Staging Classification for Carcinoma of the Stomach, 8^th^ edition. A complete pathological response (pCR) was considered when no evidence of residual tumor was found in the surgical specimen (ypT0N0). The histological grade of tumor regression was classified based on the Mandard tumor regression grade (TRG) 12.

### Postoperative management and Follow-up

Postoperative adjuvant chemotherapy was recommended for all patients included in the present study. The regimens of postoperative adjuvant chemotherapy were consistent with the preoperative part of treatment. However, some patients declined postoperative chemotherapy due to various reasons. All the patients were followed up every 3 months for the first 3 years and every 6 months afterwards until 5 years post-surgery. The follow-up content included physical examination, complete blood count, hepatic function, serum tumor marker assessment, and CT-scan as well as annual gastrointestinal endoscopy.

### Propensity score matching

In an attempt of reducing selection bias and confounding, two sets of cohorts (NAT-Surgery vs. Surgery-ACT, and nCT vs. nCRT) were created using PSM methods at a ratio of 1:1. Propensity scores were estimated using a logistic regression model and including the following characters: age, gender, year of diagnosis, tumor location, grade, clinical T-stage, clinical N-stage, clinical TNM stage (AJCC, 8^th^ edition). PSM was performed using SPSS v25 (IBM Corp., Chicago, IL, USA) with a caliper size of 0.02.

### Statistics

Categorical variables were expressed as numbers and percentages, and continuous variables were expressed as the mean ± standard deviation (SD). The Kaplan-Meier method was used to analyze the survival data, the log-rank test to compare the survival rates. The OS was calculated from the date of surgery to the date of death or last contact. The DFS was defined as the time from surgery to the date of recurrence or metastasis. The LRFS was defined as the time from surgery to the date of local recurrence. The statistical analysis was performed with SPSS v25 (IBM Corp., Chicago, IL, USA) and Graphpad Prism 7 (GraphPad Software, San Diego, California, USA). A *P* < 0.05 was considered to be statistically significant.

## Results

### Patients' characteristics

**Figure 1:**
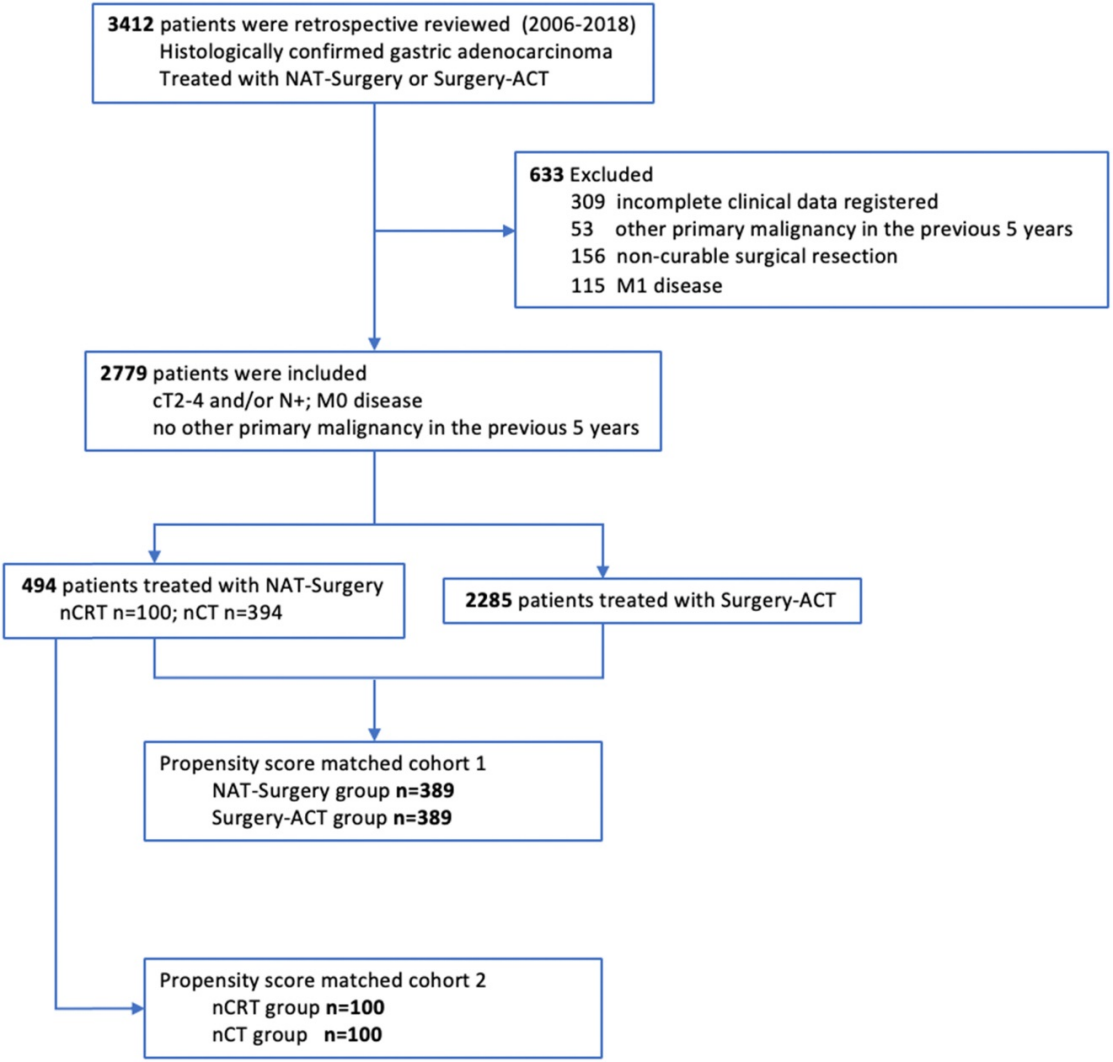
Patient Flowchart.

A total of 2779 patients were identified and included in this study (**Figure 1**). Patients were categorized into two groups according to the treatment strategies, 494 of NAT-Surgery group and 2285 of Surgery-ACT group. Patients' characteristics and tumor baseline parameters were shown in Supplement Table 1. Tumor location, grade, clinical T stage, clinical N stage and clinical TNM stage (AJCC 8^th^ edition) were significantly different between the two groups. We conducted two sets of propensity score matching (NAT-Surgery vs. Surgery-ACT and nCRT vs. nCT) to balance the baseline differences between groups for outcome comparison (**Table 1**).

### Long-term survival comparison between NAT-Surgery and Surgery-ACT

**Figure 2:**
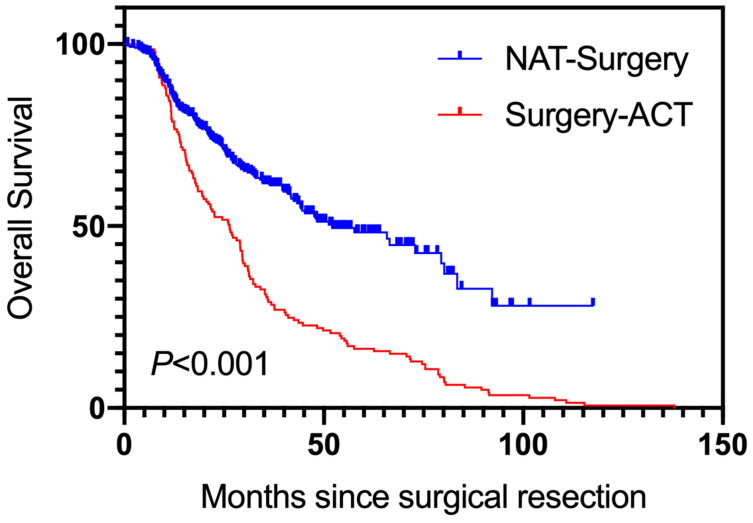
OS by treatment group in LAGC patients. NAT, neoadjuvant therapies; ACT, adjuvant chemotherapy.

After PSM, 389 pairs of patients were successfully matched (**Table 1**). Over a median follow-up of 47.0 months (range, 0.6-137.8 months), the median OS for patients in NAT-Surgery group was 52.0 months and for Surgery-ACT group was 26.4 months. The patients in NAT-Surgery group had a significant longer OS than patients in Surgery-ACT group (Hazard ratio [HR], 0.45; 95% Confidential interval [CI], 0.35-0.58; *P*<0.001, **Figure 2**).

### Neoadjuvant therapies induced toxicity effects

To further assess the outcomes of the two neoadjuvant treatments, we conducted PSM between nCRT group and nCT group and 100 pairs of patients were matched (**Table 1**). All severe adverse events that occurred during neoadjuvant treatment were summarized in **Table 2.** In the propensity score matched analysis, 29.0% of the patients in nCRT group and 14.0% in nCT group had grade 3 to 4 neutropaenia and thrombocytopaenia during neoadjuvant treatment (*P*=0.010). There was no difference between two groups in the frequency of other adverse events regarding nausea/vomiting, gastrointestinal symptoms, anemia, dyspepsia, infection, gastrointestinal bleeding, hand-foot syndrome and gastritis/esophagitis. In total, grade 3 or 4 adverse events were more frequently observed in nCRT group during neoadjuvant treatment (52.0% in nCRT group vs. 34.0% in nCT group, *P*=0.010).

### Surgical outcomes comparison between nCRT and nCT

Surgical outcomes and postoperative complications were listed in **Table 3.** The mean blood loss of nCRT group was 108 mL less than that of nCT group (170 vs. 278 mL, *P*<0.001). Moreover, the intraoperative blood transfusion rate of nCRT group was lower than that of nCT group (15.0 vs. 29.0%, *P*=0.017) and the mean postoperative hospital stay was also shorter in nCRT group (11 vs. 12 days, *P*=0.005). However, there was no difference in mean surgical time between these two groups. The total medical expenditure was comparable between the two groups (*P*=0.873).

Postoperative mortalities and morbidities were presented in **Table 3.** One patient in nCT group died within 30 days after surgical resection. Totally, the rate of postoperative complications was 9.0% and 16.0% for nCRT and nCT group (*P*=0.134), respectively.

### Pathological assessment

The pathological outcomes were shown in **Table 4.** According to the Mandard TRG, the pathological response rate (TRG1-3) was 86.0% in nCRT group versus 49.0% in nCT group (*P*<0.001). pCR was significantly increased in nCRT group than nCT group (17.0% vs. 4.0%, *P*<0.001). Moreover, in nCRT group, significantly lower ypT stage (*P*=0.002), ypN stage (*P*<0.001) and lower ypTNM stage (*P*<0.001) were observed than in nCT group. The R0 resection rate and the number of resected lymph nodes were comparable in the two groups.

### Subgroup Survival analysis of nCRT vs. nCT

**Figure 3:**
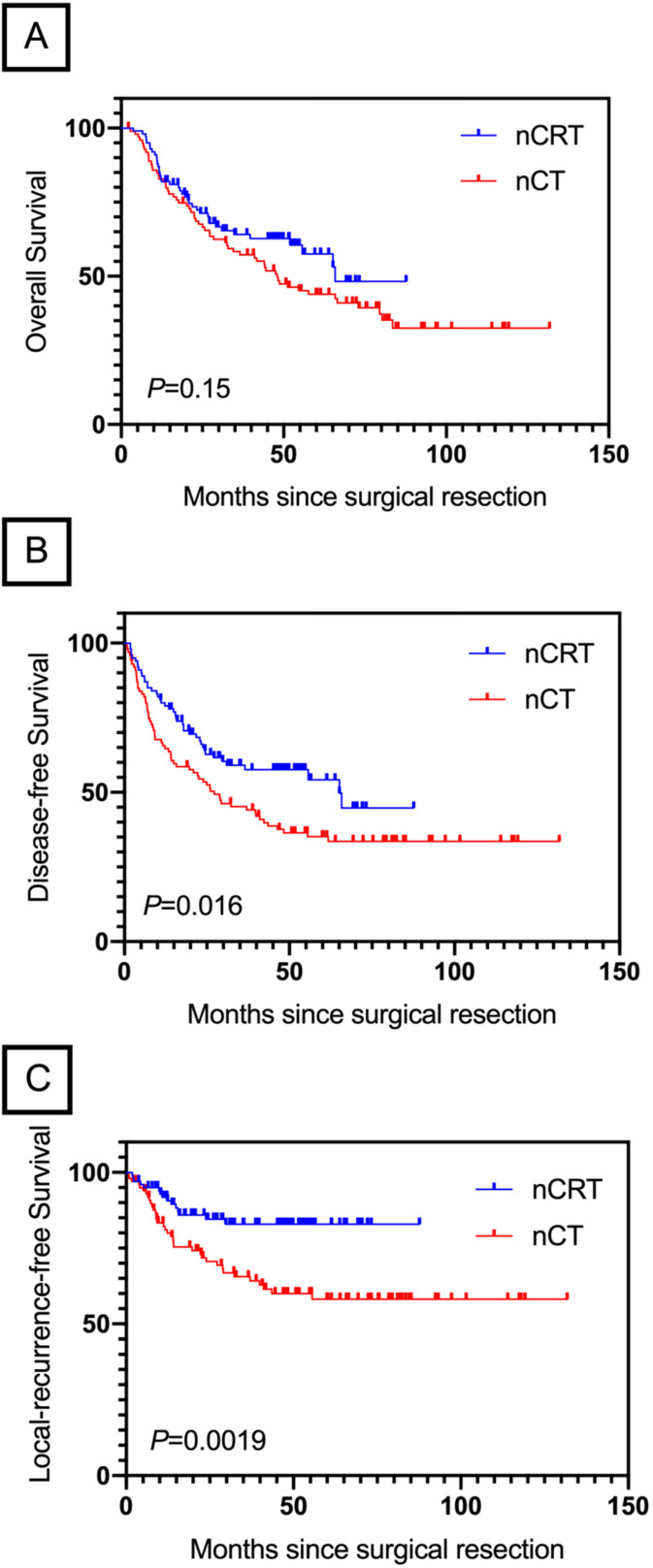
OS (A), DFS (B) and LRFS (C) by treatment group in LAGC patients underwent NAT. nCRT, neoadjuvant chemoradiation; nCT, neoadjuvant chemotherapy.

After a median follow-up of 39.2 months (range, 2.2-131.8 months), median OS in nCRT group and nCT group was 65.5 and 48.2 months, respectively. There was no difference in OS between these two groups (HR, 0.45; 95% CI, 0.51- 1.11; *P*=0.15; Figure 3A). Patients in nCRT group showed an improvement in DFS (HR, 0.63; 95% CI, 0.43-0.92; *P*=0.014; Figure 3B) and LRFS (HR, 0.40; 95% CI, 0.23-0.69; *P*=0.0019; Figure 3C) than patients in nCT group.

## Discussion

Comparing with surgery alone, survival benefits of neoadjuvant or adjuvant treatments combined with radical surgery for LAGC were shown by several RCTs 3, 6-9, 13. However, evidence from head to head trails for comparing these two therapeutic concepts was limited or unavailable. The present study had shown a survival advantage of NAT-Surgery compared to Surgery-ACT. The results from our study support the use of neoadjuvant treatments instead of undergoing radical surgery in the first place for LAGC patients. The possible explanation was that preoperative treatments could downstage the tumor, improve pathological responses, eradicate microscopic disease, reduced the risk of local and distant relapses, thus improving the OS 2.

Preoperative chemoradiation has now become the standard of care for patients with oesophageal and gastroesophageal junction tumors, thereby lending further support to its use in gastric cancer 14. However, preoperative chemoradiotherapy in LAGC patients is still considered investigational. According to our results, the addition of radiotherapy to neoadjuvant chemotherapy significantly increased the pathological response rate (86.0% vs. 49.0%, *P*<0.001) and pCR rate (17.0% vs. 4.0%, *P*<0.001). These data consisted with the previous results from several studies suggested a superior regression grade rate after preoperative chemoradiation in comparison with that seen with chemotherapy alone 15-17. Moreover, nCRT was also correlated with a lower ypT, ypN and ypTNM stage then nCT group in the current study. This suggests that chemoradiation therapy induced a higher response rate not only in the tumor wall but also in the lymph nodes, which is also consistent with conclusion of a previous study that preoperative chemoradiation increases the likelihood of achieving favorable histopathological features 16.

In the present study, we found that nCRT achieved significantly better DFS (*P*=0.014) and LRFS (*P*=0.0019) than nCT. These results support the current hypothesis that adding chemoradiation to standard perioperative chemotherapy will achieve even greater survival gains in LAGC patients. To our knowledge, the improvement for local disease control and distant recurrence after adding radiation to neoadjuvant therapy in LAGC patients has not been described previously. A possible reason for this difference could be that radiation eradicates the microscopic disease and malignant cells in the irradiated volume are radiosensitised and microscopic deposits outside are treated by adding chemotherapy 5.

The OS benefits of the use of nCRT in LAGC patients' remain controversial. Several studies have shown that the addition of radiation to neoadjuvant chemotherapy does not affect the OS 17-19. On the other hand, several researches reported a survival advantage for preoperative chemotherapy compared with preoperative chemotherapy for adenocarcinoma of EGJ 20, 21. Although, in the current study, there was no difference in OS between nCRT and nCT group, the analysis of survival revealed a type revealed a trend towards improved survival after the addition of radiotherapy among LAGC patients. This fact may be partly attributable to the relatively short follow-up period for the group of nCRT.

One of the major arguments with the use of nCRT is toxicity. Although, in the present study, the rate of grade 3 or 4 adverse events was significantly higher in nCRT group (52.0% vs. 34.0%, *P*=0.010), no death event during neoadjuvant treatment was recorded in both nCRT and nCT group. It was believed that nCRT could increase the difficulty of surgery and postoperative complications 21, 22. Interestingly, the current study revealed a less mean blood loss, lower the intraoperative blood transfusion rate and shorter mean postoperative hospital stay in nCRT group. Moreover, the postoperative complications in both groups were comparable. Nevertheless, these data should be viewed with caution, given its retrospective exploratory nature and the limited number of patients analyzed.

Strengths and limitations should be considered when interpreting the study results. The present study was a retrospective cohort study using PSM method, which minimized the potential selection bias and confounding. Our study, for the first time, reported that nCRT had survival benefits of DFS and LRFS compared with nCT among LAGC patients. However, some patients were missing crucial clinical data that may have also affected survival outcomes such as comorbidities. Moreover, our follow-up was relatively short. Finally, this was a single-institutional analysis with significant treatment heterogeneity.

## Conclusion

In conclusion, NAT improved survival outcomes among LAGC patients over surgery followed by adjuvant chemotherapy. In comparison with nCT, nCRT resulted in higher pCR rate, better DFS and LRFS, without significantly affecting OS.

## Supplementary Material

Supplementary table S1.

## Figures and Tables

**Table 1:** Baseline characteristics of the two sets of propensity scoring matched cohort. (1)NAT-Surgery vs. Surgery-ACT; (2) nCRT vs. nCT

Characteristics	NAT-Surgery	Surgery-ACT	*P* value
	n=389 (%)	n=389 (%)	
**Age**			
mean (±SD)	56.13 (±10.42)	55.94 (±10.34)	0.992
**Gender**			
Female	91 (23.4)	107 (27.5)	
Male	298 (76.6)	282 (72.5)	0.181
**Tumor location**			
Upper stomach	129 (33.2)	89 (22.9)	
Lower stomach	215 (55.3)	279 (71.7)	
Whole stomach	45 (11.6)	21 (5.4)	<0.001
**Grade**			
Well differentiated	8 (2.1)	5 (1.3)	
Moderately differentiated	90 (23.1)	116 (29.8)	
Poor differentiated	291 (74.8)	268 (68.9)	0.068
Clinical T Stage*			
T2	24 (6.2)	20 (5.1)	
T3	121 (31.1)	92 (23.7)	
T4	244 (62.7)	277 (71.2)	0.037
Clinical N stage*			
cN0	37 (9.5)	45 (11.6)	
cN-positive	352 (90.5)	344 (88.4)	0.344
CTNM Stage*			
II	61 (15.7)	71 (18.3)	
III	328 (84.3)	318 (81.7)	0.331
***Characteristics***	***nCRT***	***nCT***	***P value***
	***n=100 (%)***	***n=100 (%)***	
**Age**			
mean (±SD)	56 (±9.2)	54 (±11.6)	0.094
**Gender**			
Female	16 (16.0)	26 (26.0)	0.083
Male	84 (84.0)	74 (74.0)	
**Tumor location**			
Upper stomach	59 (59.0)	59 (59.0)	0.5
Lower stomach	36 (36.0)	36 (36.0)	
Whole stomach	5 (5.0)	5 (5.0)	
**Grade**			
Well differentiated	4 (4.0)	2 (2.0)	0.488
Moderately differentiated	17 (17.0)	13 (13.0)	
Poor differentiated	79 (79.0)	85 (85.0)	
Clinical T stage*			
T2	1 (1.0)	1 (1.0)	0.539
T3	24 (24.0)	31 (31.0)	
T4a	75 (75.0)	68 (68.0)	
Clinical N stage*			
cN0	1 (1.0)	6 (6.0)	0.124
cN-positive	99 (99.0)	94 (94.0)	
Clinical TNM Stage*			
II	10 (10.0)	7 (7.0)	0.447
III	90 (90.0)	93 (93.0)	

* Tumor stage according to the American Joint Committee on Cancer, 8th Edition. NAT, neoadjuvant therapies; ACT, adjuvant chemotherapy; nCRT, neoadjuvant chemoradiation; nCT, neoadjuvant chemotherapy; SD, standard deviation.

**Table 2:** Severe adverse events between two neoadjuvant treatment groups after propensity score matching

Characteristic	nCRT, n=100 (%)	nCT, n=100 (%)	*P* value
**Grade 3 or 4 adverse events during neoadjuvant treatment**			
Nausea and vomiting	7 (7.0)	6 (6.0)	0.774
Gastrointestinal symptoms	7 (7.0)	7 (7.0)	1.000
Neutropaenia/Thrombocytopaenia	29 (29.0)	14 (14.0)	0.010*
Anemia	1 (1.0)	1 (1.0)	1.000
Dyspepsia	4 (4.0)	3 (3.0)	1.000
Infection	0 (0.0)	2 (2.0)	0.477
Gastrointestinal bleeding	0 (0.0)	1 (1.0)	0.238
Hand-foot syndrome	0 (0.0)	0 (0.0)	1.000
Gastritis or Esophagitis	4 (4.0)	0 (0.0)	0.130
Total number of SAEs	52 (52.0)	34 (34.0)	0.010*

nCRT, neoadjuvant chemoradiation; nCT, neoadjuvant chemotherapy; SAE, severe adverse events.

**Table 3:** Surgical outcomes and postoperative complications of two neoadjuvant groups after propensity score matching

Characteristic	nCRT n=100 (%)	nCT n=100 (%)	*P* value
**Estimated blood loss, ml**			
mean (±SD)	170 (± 157.8)	278 (±218.2)	**<0.001**
**Surgical time, minutes**			
mean (±SD)	200 (± 51.7)	205 (±60.8)	0.497
**Intraoperative blood transfusion**			
	15 (15.0)	29 (29.0)	**0.017**
**Length of postoperative hospital stay**			
mean (±SD)	11 (± 3.6)	12 (±3.8)	**0.005**
**Medical expenditure**			
mean (±SD, yuan)	201574.33 (± 33413.76)	203647.28 (± 49283.49)	0.873
**Postoperative complications**			
30-day mortality	0 (0.0)	1 (1.0)	0.238
Abdominal infection	2 (2.0)	4 (4.0)	0.678
Anatommotic leakage	2 (2.0)	2 (2.0)	1.000
Anatommotic stenosis	2 (2.0)	0 (0.0)	0.477
Wound infection	2 (2.0)	4 (4.0)	0.678
Abdominal bleeding	2 (2.0)	2 (2.0)	1.000
Pneumonia	1 (1.0)	1 (1.0)	1.000
Cardiovascular complications	0 (0.0)	1 (1.0)	0.238
Postoperative gastrointestinal dysfunction	1 (1.0)	1 (1.0)	1.000
Rental failure	0 (0.0)	0 (0.0)	1.000
Total number of postoperative complications	9 (9.0)	16 (16.0)	0.134

nCRT, neoadjuvant chemoradiation; nCT, neoadjuvant chemotherapy; SD, standard deviation.

**Table 4:** Pathological outcomes of neoadjuvant groups after propensity score matching

Characteristic	nCRT n=100 (%)	nCT n=100 (%)	*P* value
Tumor regression grade*			
Complete response	17 (17.0)	4 (4.0)	**<0.001**
Partial response	69 (69.0)	45 (45.0)	
No response	14 (14.0)	51 (51.0)	
**R0 resection**	96 (96.0)	89 (89.0)	0.060
**ypT Stage#**			
ypT0	17 (17.0)	4 (4.0)	**0.002**
ypT1	12 (12.0)	6 (6.0)	
ypT2	23 (23.0)	16 (16.0)	
ypT3	20 (20.0)	25 (25.0)	
ypT4a	25 (25.0)	39 (39.0)	
ypT4b	3 (3.0)	10 (10.0)	
**ypN Stage#**			
ypN0	59 (59.0)	25 (25.0)	**<0.001**
ypN1	24 (24.0)	21 (21.0)	
ypN2	8 (8.0)	22 (22.0)	
ypN3	9 (9.0)	32 (32.0)	
**ypTNM Stage#**			
pCR	17 (17.0)	4 (4.0)	**<0.001**
I	28 (28.0)	13 (13.0)	
II	27 (27.0)	22 (22.0)	
III	28 (28.0)	61 (61.0)	
**Lymph nodes resected**			
1-10	5 (5.0)	7 (7.0)	0.430
11-20	22 (22.0)	31 (31.0)	
21-30	42 (42.0)	36 (36.0)	
>30	31 (31.0)	26 (26.0)	

*Tumor regression grade according to Mandard tumor regression grade, TRG1 (pathological complete regression) was defined as complete response, TRG2 and TRG3 were defined as partial response, TRG4 and TRG5 were defined as no response. #Tumor stage according to the American Joint Committee on Cancer, 8th Edition. Abbreviations: nCRT, neoadjuvant chemoradiation; nCT, neoadjuvant chemotherapy.
