# Supplementary material for: The Effect of Neoadjuvant Therapies for Patients with Locally Advanced Gastric Cancer: A Propensity Score Matching Study
Source: J Cancer. 2021 Jan 1;12(2):379–86. doi: 10.7150/jca.46847 (PMC7738980; doi:10.7150/jca.46847)
Supplement: Supplementary file 1 — Supplementary table S1. [file jcav12p0379s1.pdf]

Supplement Table 1. Baseline characteristics of two different types of therapeutic strategies for LAGC.

Characteristics		NAT-Surgery n=494 (%)	Surgery-ACT n=2285 (%)	***P***
**Age**				
	mean (±SD)	55.58(±10.62)	56.21(±10.30)	0.152
**Gender**				
	Female	132 (26.7)	559(24.5)	
	Male	362 (73.3)	1726(75.5)	0.293
**Tumor location**				
	Upper stomach	129 (26.1)	862(37.7)	
	Lower stomach	221 (44.7)	1361(59.6)	
	Whole stomach	144 (29.1)	62(2.7)	<0.001
**Grade**				
	Well differentiated	9 (1.8)	57(2.5)	
	Moderately differentiated	95 (19.2)	941(41.2)	
	Poor differentiated	390 (78.9)	1287(56.3)	<0.001
**Clinical T Stage***				
	T2	17 (3.4)	205(9.0)	
	T3	151 (30.6)	1017(44.5)	
	T4a	326 (66.0)	1063(46.5)	<0.001
**Clinical N Stage***				
	cN0	37 (7.5)	350(15.3)	
	cN-positive	457 (92.5)	1935(84.7)	<0.001
**Clinical TNM Stage***				
	II	61 (12.3)	676(29.6)	
	III	433 (87.7)	1609(70.4)	<0.001
**NAT Strategies**				
	nCRT	100 (20.2)	-	
	nCT	394 (79.8)	-	

* Tumor stage according to the American Joint Committee on Cancer, 8th Edition.

NAT, neoadjuvant treatment; ACT, adjuvant chemotherapy; SD, standard deviation.
